# Adversarial Attacks on Medical Image Classification

**DOI:** 10.3390/cancers15174228

**Published:** 2023-08-23

**Authors:** Min-Jen Tsai, Ping-Yi Lin, Ming-En Lee

**Affiliations:** Institute of Information Management, National Yang Ming Chiao Tung University, Hsin-Chu 300, Taiwan

**Keywords:** machine learning, artificial intelligence, adversarial learning, computer vision, metaheuristic

## Abstract

**Simple Summary:**

As we increasingly rely on advanced imaging for medical diagnosis, it’s vital that our computer programs can accurately interpret these images. Even a single mistaken pixel can lead to wrong predictions, potentially causing incorrect medical decisions. This study looks into how these tiny mistakes can trick our advanced algorithms. By changing just one or a few pixels on medical images, we tested how various computer models handled these changes. The findings showed that even small disruptions made it hard for the models to correctly interpret the images. This raises concerns about how reliable our current computer-aided diagnostic tools are and underscores the need for models that can resist such small disturbances.

**Abstract:**

Due to the growing number of medical images being produced by diverse radiological imaging techniques, radiography examinations with computer-aided diagnoses could greatly assist clinical applications. However, an imaging facility with just a one-pixel inaccuracy will lead to the inaccurate prediction of medical images. Misclassification may lead to the wrong clinical decision. This scenario is similar to the adversarial attacks on deep learning models. Therefore, one-pixel and multi-pixel level attacks on a Deep Neural Network (DNN) model trained on various medical image datasets are investigated in this study. Common multiclass and multi-label datasets are examined for one-pixel type attacks. Moreover, different experiments are conducted in order to determine how changing the number of pixels in the image may affect the classification performance and robustness of diverse DNN models. The experimental results show that it was difficult for the medical images to survive the pixel attacks, raising the issue of the accuracy of medical image classification and the importance of the model’s ability to resist these attacks for a computer-aided diagnosis.

## 1. Introduction

The use of ML models in the medical domain enables AI to assist doctors in hospitals as a critical diagnostic tool. ML is utilized widely in healthcare for its power to assist in the early diagnosis of diseases reasonably quickly and accurately. The ML model can provide a quick diagnosis based on medical images due to the development of deep learning in the medical field. Although ML is shown to have remarkable performance in various domains, many researchers have found ML models to be susceptible to different types of adversarial attacks. For example, Mahmood et al. [1] demonstrated adversarial attacks taking place in the physical realm with eyeglass frames designed to fool face-recognition systems, and [2] proposed that an adversarial attack can reprogram neural networks to perform novel adversarial tasks.

Real-life adversarial attacks on deep learning models pose significant concerns due to their potential danger. In a notable study, Eykholt et al. [3] demonstrated the feasibility of robust physical-world attacks by perturbing a real stop sign using black and white stickers, resulting in targeted misclassification. These attacks are particularly concerning due to their ability to withstand various physical conditions, including changes in view angles, distances, and resolutions. This highlights the potential for adversaries to use deep learning models to manipulate real-world objects, such as traffic signs, leading to misperception. Such attacks have critical implications for safety and security, as they can deceive autonomous systems, including self-driving cars, and cause dangerous consequences on roads.

Real-life adversarial attacks on deep learning models not only pose significant concerns, but they also have potentially life-threatening consequences, particularly in the medical field. A misclassified medical image due to an attack could lead to delayed or incorrect treatment, potentially endangering patients’ lives. Acknowledging the criticality of securing ML models in the medical domain and developing robust defense mechanisms to mitigate the risks associated with adversarial attacks is crucial. Ensuring the integrity and reliability of AI-powered diagnostic tools is paramount to safeguarding patients’ well-being and providing accurate medical care.

This study uses a one-pixel attack on various medical images and a multi-pixel attack on the COVID-19 chest X-ray dataset. These datasets contain multiclass and binary-class multi-label classifications. According to the experiment results, most medical images could be perturbed into adversarial images. As the original one-pixel attack paper [4] did not cover the topic of multi-labels, a method was devised to evaluate the effectiveness of a multi-label dataset attack. The overall contribution of this study is as follows:

- Test the effectiveness of a pixel attack on various types of medical images;
- Develop an effective way to evaluate the effectiveness of attacks on multi-label datasets.

## 2. Related Work

Most adversarial attacks can currently be divided into the following categories:

### 2.1. White Box Attack

Szegedy et al. [5] were the first to show that an adversarial attack can cause the network to misclassify an image based on applying hardly perceptible perturbation. In a white box attack, the attacker needs access to the victim model, including its structure, training datasets, or parameters. Goodfellow et al. [6] proposed a fast gradient sign method (FGSM) that can quickly generate adversarial samples without hyperparameter tuning or additional processing.

### 2.2. Black Box Attack

In a black box attack, the attacker does not need access to training samples and the model’s structure. The two main types of black box attacks are query-based and transfer-based. Yan et al. [7] proposed the subspace attack method, which reduces the complexity of the query by limiting the search directions of gradient estimation by promising subspaces that are spanned by the input gradients of a few reference models. Meanwhile, Dong et al. [8] proposed a translation-invariant attack method to generate adversarial examples with better transferability.

### 2.3. One-Pixel Attack

Adversarial images are generated by changing only one pixel in the original image. The original one-pixel attack paper [4] contained a simple but effective way to generate adversarial images by utilizing a different evolution algorithm to select the sensitive pixels and optimal perturbation in a CIFAR-10 dataset. To determine why a one-pixel attack may work, Danilo [9] used propagation maps that demonstrated the effect of the perturbation in each layer of the model. Moreover, he found that pixels near the perturbed one in the one-pixel attack tended to share the same susceptibility, showing that neither neurons nor pixels are the primary sources of vulnerability but rather the receptive fields. This is because most image recognition neural networks contain convoluted layers, which makes them more susceptible.

### 2.4. Adversarial Attack on Medical Images

In their study, Ma et al. [10] focused on adversarial attacks on deep learning-based medical image analysis systems. Their experiments on benchmark medical image datasets showed that adversarial attacks on medical images are easier to craft due to the unique characteristics of medical image data and deep neural network models. They also demonstrated that medical adversarial examples tend to target areas outside pathological regions, resulting in fundamentally different and deep features that are easy to separate.

In their study, Paul et al. [11] investigated the impact of adversarial attacks on the accuracy of predicting lung nodule malignancy and developed an ensemble-based defense strategy. They also explored the addition of adversarial images in the training set to enhance robustness against adversarial attacks. Experiments conducted on a subset of cases from the National Lung Screening Trial (NLST) dataset revealed that adversarial attacks, specifically the Fast Gradient Sign Method (FGSM) and one-pixel attacks, significantly affected the accuracy of CNN predictions. They found that a defense strategy involving multi-initialization ensembles and training with adversarial images can improve classification accuracy.

Ozbulak et al. [12] investigated the impact of adversarial examples on deep learning models to segment medical images with a specific focus on skin lesion and glaucoma optic disc segmentation and introduced the Adaptive Mask Segmentation Attack, a novel algorithm capable of generating adversarial examples with realistic prediction masks. This algorithm is based on utilizing perturbations that are mainly imperceptible to the human eye but lead to misclassification. This research sheds light on the potential risks associated with adversarial examples in segmenting medical images and highlights the need for robust defenses to ensure the reliability and accuracy of segmentation models used in clinical applications.

## 3. Approach

### 3.1. One-Pixel Attack

Considering that the original image could be represented by an *n*-dimensional array $x=\left(x_{1},x_{2},\ldots ,x_{n}\right)$, *f* is the model we chose to attack. The input of model *f* is the original image *x*, from which the confidence level of what category *x* is could be obtained, which is *f(x*). The adversarial image is generated by perturbing a pixel in the original image *x*. Here, the perturbed pixel is defined as $e\left(x\right)=\left(e_{1},e_{2},\ldots ,e_{n}\right)$ and the limit of the perturbation length is specified as *L*. Supposing that the class set in the dataset is $C=\left(c_{1},c_{2},\ldots ,c_{n}\right),$ the original image belongs to class *c_ori_*, and we want to change it into an adversarial class *c_adv_*. *c_adv_* and *c_ori_* ∈ *C*, hence this can be done using the following equation:

(1) $$\;\;\;\;\;\;\;\;\;\;\;\;\;\;\;\underset{e{\left(x\right)}^{*}}{\max}f_{c_{adv}}\left(x+e\left(x\right)\right)\;subject\;to\;{\left\|e\left(x\right)\right\|}_{0}\leq L$$

In a one-pixel attack scenario, since we only want to change one pixel, the value of *L* is set to 1. The most direct way to find the best solution is an exhaustive search, which involves trying every different pixel in the image. For a 224×224 RGB image, there will be as many as 224×224×256×256×256=841,813,590,016 possibilities. As a result, a more effective way to simulate adversarial attacks is a differential evolution.

### 3.2. Differential Evolution

**Figure 1:**
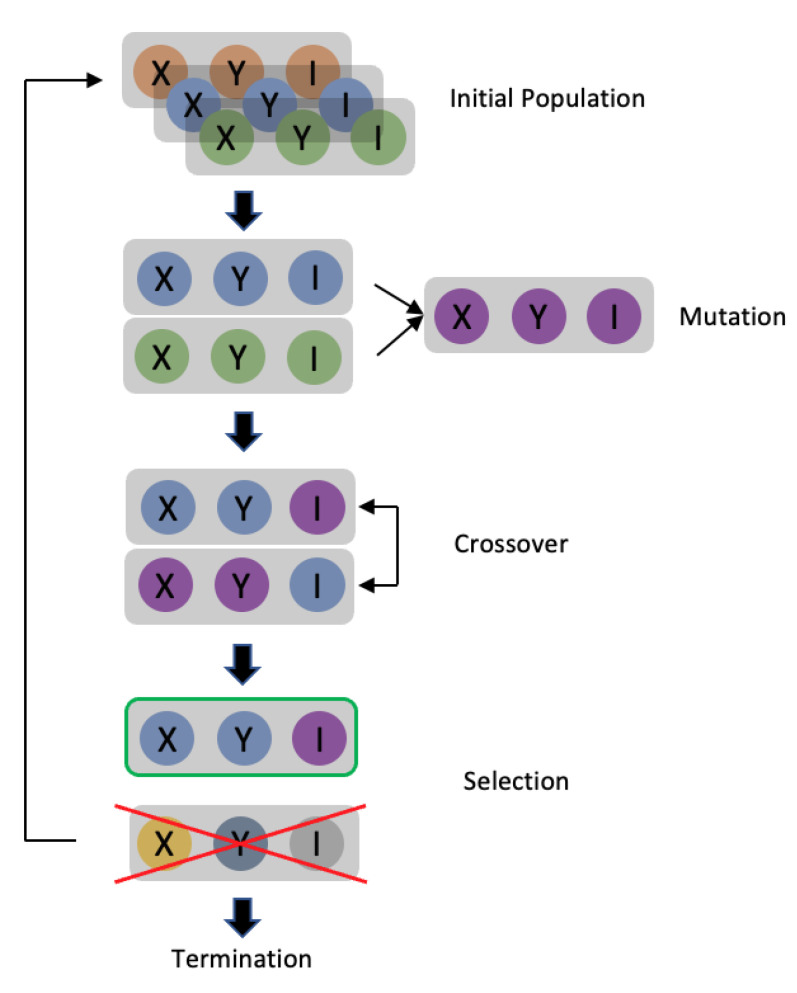
Overview of differential evolution procedure.

A differential evolution (DE) [13] is a branch of an evolution strategy (ES) [14]. The algorithm is developed by mimicking the natural breeding process. The DE process is shown in Figure 1 with each stage described as follows:

(1) Initial populations

The process starts with the generation of possible solutions to the issue to be solved. Each potential solution is called a “gene”. A set of solutions is produced in each “generation”, which is the process of a specific run of the ES. This set of solutions is called a “population”. As mentioned above, *f* is the model to be attacked for the base image *x*. In a one-pixel attack, the solution is in the form of (*X*, *Y*, *R*, *G*, *B*) array if the base image is colored, or the form of (*X*, *Y*, *I*) array if the image is a greyscale one. *X* denotes the value of the *x*-coordinate, *Y* denotes the value of the y-coordinate, and *I* denotes the value of the grey level. The solution for the two-pixel attacks and three-pixel attacks is (*X*_1_, *Y*_1_, *I*_1_, *X*_2_, *Y*_2_, *I*_2_) and (*X*_1_, *Y*_1_, *I*_1_, *X*_2_, *Y*_2_, *I*_2_, *X*_3_, *Y*_3_, *I*_3_), respectively (greyscale image). The population size is set to 100, meaning that there will be 100 adversarial arrays in each generation of the DE. The initial population will be developed randomly, after which a set of parental adversarial arrays will be set $ARR^{j}=\left(arr_{1}^{j},arr_{2}^{j},\ldots ,\;arr_{100}^{j}\right).$ The superscript indicates the number of generations, and the subscript indicates the index.

(2) Mutation

The following formula was used to generate new genes in the mutation process:

(2) $$arr_{i}^{j^{\prime }}=arr_{r_{1}}^{j}+F\cdot \left(arr_{r_{2}}^{j}-arr_{r_{3}}^{j}\right)$$

$arr_{i}^{j}$ means that this is the j generation array with index i, and the apostrophe means that this is the offspring population. r is a random number ranging between 1 and the size of the parent population. *F* is the mutant factor that ranges from 0 to 1 and decides the strength of the mutation. According to the above formula, the mutant gene firstly comprises a random parental gene $arr_{r_{1}}^{j}$, and secondly, the difference between the two parental genes $\left(arr_{r_{2}}^{j},\;arr_{r_{3}}^{j}\right)$. The mutant factor decides how much the difference between the two random parental genes will affect the “base gene” $arr_{r_{1}}^{j}$. The offspring population is generated by repeating the above equation 100 times. Assuming that this is generation j in the DE process, the generated offspring population is denoted as $ARR^{j^{\prime }}=\left(arr_{1}^{j^{\prime }},arr_{2}^{j^{\prime }},\ldots ,\;arr_{100}^{j^{\prime }}\right)$.

(3) Crossover

Since the original one-pixel attack did not include a crossover, it was not used in this work.

(4) Selection

Unlike many other evolution strategies that enable the next generation of top performance genes to survive, DE uses a pairwise survival strategy to select the group of genes that will survive. The selection process will be applied to each parent and offspring pair. There will now be two sets of arrays in our work: $ARR^{j}$ and $ARR^{j^{\prime }}$, each of which contains 100 arrays in the form of (*X*, *Y*, *I*) (for the one-pixel attack on a grayscale image). Each array will generate a corresponding adversarial image modified from the original image. Hence, the algorithm will now have two groups of adversarial images $X^{j}=\left(X_{1}^{j},X_{2}^{j},\ldots ,\;X_{100}^{j}\right)$ and $X^{j^{\prime }}=\left(X_{1}^{j^{\prime }},X_{2}^{j^{\prime }},\ldots ,\;X_{100}^{j^{\prime }}\right)$. These images will then be input to trained model *f* to generate two sets of confidence level arrays, $CL^{j}=\left(cl_{1}^{j},cl_{2}^{j},\ldots ,\;cl_{100}^{j}\right)$ and $CL^{j^{\prime }}=\left(cl_{1}^{j^{\prime }},cl_{2}^{j^{\prime }},\ldots ,\;cl_{100}^{j^{\prime }}\right)$. The performance of the adversarial images can be evaluated based on the confidence level. Supposing that the class set in the dataset $C=\left(c,c_{2},\ldots ,c_{n}\right)$ and the original image belongs to class *c_ori_.*, the confidence level arrays that are generated can be denoted as $cl_{i}^{j}=\left(cl_{i_{1}}^{j},cl_{i_{2}}^{j},\ldots ,\;cl_{i_{100}}^{j}\right),\;cl_{i_{k}}^{j}\in \left[0,\;1\right],\;\sum_{k=1}^{n}\;cl_{i_{k}}^{j}=1$. Each element of each confidence level array corresponds to the confidence level of the class. $cl_{i_{k}}^{j}$ is how confident the model is that the image belongs to class *c_k_*. The groups of adversarial arrays that survive will then be selected when the algorithm pairs the confidence level arrays in an equation. It will compare each group of confidence levels, which means it will compare $\left(cl_{i}^{j},\;cl_{i}^{j^{\prime }}\right)$ on the kth position of the confidence level array. Supposing that *c_k_* is the target class, the target attack experiment aims to maximize the fitness score. This means that the confidence level with the higher value should be reserved. For instance, the parental gene $arr_{i}^{j}$ will perform better if $cl_{i}^{j}<cl_{i}^{j^{\prime }}$. Notably, the algorithm will preserve the parental gene when the performance of both genes is similar. This group of preserved genes is then passed to the next step.

(5) Termination

An early-stop mechanism is established to determine if the performance is good enough. Based on the above selection process, the algorithm has 100 adversarial arrays corresponding to 100 adversarial images, each belonging to a specific class in class set C. In a non-target attack, the process will be terminated if one image has a class that is different from the original image. On the other hand, the process will be terminated in a targeted attack if one image has the same class as the target class; otherwise, the preserved group of genes will become the new parental initial population, and the DE process will be re-run. The process will also be terminated when the maximum iteration is reached.

### 3.3. One-Pixel Attack Fitness Score Setting on a Multiclass Dataset and Multi-Label Dataset

Only multiclass datasets were used in the original one-pixel attack paper, but in medical images one image could contain multiple diseases, making a multi-label classification problem. The classifier not only needs to determine whether the image is diseased or not, but it also needs to identify all the diseases in the image. Recalling that a one-pixel attack uses DE to generate the adversarial images, and DE requires a fitness score to assess the performance of the generated images, the target or original class confidence level was used as the fitness score in the original one-pixel attack study. Suppose that the class set in the dataset is $C=\left(c_{1},c_{2},\ldots ,c_{n}\right)$, and the original image belongs to the class $c_{ori}$. $c_{ori}\in C$, hence when this image is processed by the classifier it will generate a confidence interval vector $cl=\left(cl_{1},cl_{2},\ldots ,cl_{n}\right),\;cl_{i}\in \left[0,1\right],\;\overset{n}{\underset{i=1}{\sum }}cl_{i}=1$, and $cl_{ori}$ will denote the confidence level of the original class. $cl_{ori}\in cl$. If the experiment involves conducting a non-target attack, the goal will be to minimize $cl_{ori}$. If the experiment involves conducting a targeted attack and the adversarial images need to become class $c_{adv}$ ($c_{adv}\in \;C$), the goal will be to maximize the confidence level of class $cl_{adv}$. $cl_{adv}\in C$. The same technique will be used for the multiclass datasets in this study [8], but for the multi-label datasets the algorithm may not look at just one specific class. It will look at multiple class confidence levels at once. Supposing that the class set in the dataset is $C=\left(c_{1},c_{2},\ldots ,c_{n}\right),$ the image label can be constructed with an array $l=\left(l_{1},l_{2}\ldots ,l_{n}\right)$. $l_{i}\;\left(i\in N,\;i\in \left[0,n\right]\right)$. Each $l_{i}$ corresponds to each class $c_{i}$ in the class set C and the value will be 0 or 1. If the value is 0, the image does not contain this class, but if the value is 1 it does. The image can be considered to be a multi-dimensional array $x=\left(x_{1},x_{2},\ldots ,x_{n}\;\right)$, and the classifier can be represented by f. When the image x is input to the classifier f, $f\left(x\right)$ will produce a set of confidence levels $cl=\left(cl_{1},\;cl_{2},\ldots ,c_{ln}\right),\;cl_{i}\in \left[0,1\right],\;i\in N$. Threshold γ can be set with a range from 0 to 1, where, if $cl_{i}>\gamma$, the algorithm will consider it to contain a class $c_{i}$ disease. Therefore, if all $c_{i}<\gamma$, the image is of a normal patient with no disease. Suppose that an image $x_{ori}$ that we want to attack is found with the original class set $c_{ori}$. $c_{ori}$ is a subset of C and has a label form $l_{ori}$. $l_{ori}$ is a subset of l. By inputting the image $x_{ori}$ into the classifier it becomes $f\left(x_{ori}\right)$, and the classifier has successfully predicted the image and produced confidence level $cl_{ori}$. Because all the elements in the $c_{ori}$ needed to be considered at once, cosine similarity is used in this study to construct the fitness score. The generated adversarial image $x_{adv}$ will be input into the classifier and become $f\left(x_{adv}\right),$ hence producing an adversarial confidence level $cl_{adv}$. If this is a non-target attack, the formula will be as follows:

(3) $$\;\;\;similarity\left(l_{ori},cl_{adv}\right)=\frac{l_{ori}\cdot cl_{adv}}{\max\left(\|l_{ori}\|\|cl_{adv}\|,\delta \right)},\;\delta =10^{-5}\;\;$$

δ is a very small number that prevents the denominator from becoming 0. The goal is to minimize the above formula.

If this is the target attack and the target class set is $c_{adv}$, $c_{adv}$ is a subset of C and has a label form $l_{adv}$, $l_{adv}$ is a subset of l, the formula will be as follows:

(4) $$\;\;\;\;similarity\left(l_{adv},cl_{adv}\right)=\frac{l_{adv}\cdot cl_{adv}}{\max\left(\|l_{adv}\|\|cl_{adv}\|,\delta \right)},\;\delta =10^{-5}\;$$

The goal is to maximize the above formula. The algorithm transformed the label and confidence level range from [0, 1] to [−1, 1] as a reminder to calculate the cosine similarity. This is because if the label is all zeroes, the cosine similarity will always be zero, causing the fitness function to fail.

## 4. Experimental Setting

All experiments are non-target attack experiments except for the COVID-19 dataset. For non-target attacks, each class in each multiclass dataset will undergo 100 individual experiments. We attack all test images in each class for a targeted attack on the COVID-19 dataset. For the multi-label dataset, which is the Chest dataset, only five classes were involved in the experiment with the five larges amounts of data in the whole dataset. The reason for this will be explained in the Chest dataset section.

### 4.1. Differential Evolution

This DE process has no crossover like the original one-pixel attack paper, and the mutant factor is set to 0.5. The population size is set to 100. The maximum number of iterations to run the DE process is limited to 100. It may stop if it meets the early-stop condition, indicating that the total number of successful images has surpassed 1% of the total number of adversarial images generated in that particular iteration.

### 4.2. Model

We used a stochastic gradient descent (SGD) optimizer with a learning rate = 0.001 and momentum = 0.9. The main model was ResNet50 [15]. Each model trained 100 epochs with batch size = 64. To prevent overfitting and optimize the training efficiency, we implemented early stopping with a condition that terminates training when the model achieves the 95% accuracy threshold.

### 4.3. Hardware and Software

The CPU is 11th Gen Intel(R) Core (TM) i7-11700KF @ 3.60GHz 3.60 GHz and the RAM is 16 GB. The GPU is NVIDIA GeForce RTX 3060 and the RAM is 12 GB.

The software is the Python package “torchvision”. At the same time, the model is pretrained and adjusted in the first layer according to the dataset size and number of channels. The last layer is also adjusted based on the class number of the dataset. The software specifications are OS: Windows 10 × 64 Education. Programming language: Python 3.9.7. Programming modules: numpy 1.20.3, torchvision 0.11.3, pytorch 1.10.2, tqdm 4.62.3, scikit-learn 0.24.2, pillow 8.4.0, tiffile 2021.7.2, matplotlib 3.4.3, pandas 1.3.4, pyyaml 6.0, sqlite 3.36.0, plotly 5.8.2.

### 4.4. Dataset

The Derma, Pneumonia, and OCT datasets will be split into training and testing sets with a ratio of 8:2. The training and testing split for the COVID-19 dataset will be the same as the original setting of [16]. For the Chest dataset some of the labels in the Chest multi-label dataset contain only one piece of data, which cannot be split into training and testing sets. Instead, the provider of this dataset produced a list of training and testing images that will be used for the experiment on the Chest dataset. Multi-label datasets have a different training option from multiclass datasets.

The multiclass training set will be used to train and obtain the test accuracy. The multi-label training set will exclude all “normal” cases because a “normal” label is all zeroes, which will easily cause the model to train in the wrong direction.

The “normal” case also has the most significant data points in the dataset that will contribute to 94% accuracy of the model by predicting all cases to be “normal”. Therefore, the experiment excludes this class in order to train the model appropriately. An overview of the dataset information is provided in Table 1. The images in different datasets come in different sizes and will be resized as 224 × 224. The contents of each tested database are as follows:

(1) Derma

This is a multiclass dermatoscopic colored image dataset of common pigmented skin lesions. It consists of 10,015 images and seven categories. The dataset [17] has a size of 3 × 600 × 450. An overview of the dataset information is provided in Table 2.

(2) Pneumonia

The Pneumonia dataset, sourced from the study [18], consists of a comprehensive collection of 5856 greyscale chest X-ray images, which provide a valuable insight into a diagnosis of pneumonia. Comprised of two classes, “Normal” (1583 images) and “Pneumonia” (4273 images), this dataset provides a solid foundation for training and evaluating machine learning models in the detection and differentiation of pneumonia cases. The images will be center-cropped with a 20% reduction in width and height, effectively removing most artificial labels to ensure an accurate analysis. The precision of the “Pneumonia” class is 88%, with a recall of 100%. The precision of the “Normal” class is 100%, with a recall of 65%.

(3) OCT

The Optical Coherence Tomography (OCT) dataset, which is referenced in [18], is a large and diverse collection of medical imaging data specifically designed for the study and analysis of retinal diseases. It contains 109,309 high-resolution grayscale images. The dataset is organized into four distinctive classes, each of which represents a specific retinal disease or condition: “Normal” (51,390 images), “Choroidal neovascularization” (37,455 images), “Diabetic macular edema” (11,598 images), and “Drusen” (8866 images). The precision of the “Normal” class is 97%, with a recall of 100%. The precision of the “Choroidal neovascularization” class is 97% with a recall of 97%. The precision of the “Diabetic macular edema” class is 95% with a recall of 93%, and the precision of the “Drusen” class is 92% with a recall of 79%.

(4) COVID-19 Chest X-ray

This public COVID-19 chest X-ray dataset [16] consists of images and other associated metadata, including patients’ age, gender, etc. The latest V9A version of the training and testing dataset was used. This contains 30,130 training images and 400 testing images across three classes: normal, pneumonia, and COVID-19 cases. The same training and testing split as the original setting of [16] was used in the experiment, and the image distribution is shown in Table 3.

(5) Chest

The Chest dataset is a binary-class multi-label frontal-view X-ray chest greyscale image dataset with a total of 112,120 images and 14 classes of disease. The dataset is taken from the NIH-Chest X-ray 14 dataset [19]. The original size of the images is 1 × 1024 × 1024. The labels contained in the dataset are shown in Table 4. For example, an image with the label [0, 1, 1, 0, 0, 0, 0, 0, 0, 0, 0, 0, 0, 0] shows that this is an image with the labels Cardiomegaly and Effusion. If we want to perform each possible combination, there will be $2^{14}=16,384$ possible combinations considering there are 14 classes in the dataset. This will mean conducting 1,638,400 experiments only on the Chest dataset. However, the dataset only contains 247 different combinations of labels, most of which have a total number of images under 100, and even under 10. To prevent the model from not classifying certain types of images due to a lack of training images, we only chose the five combinations of labels with the most data in the dataset: Normal with 60,361 images, Infiltration with 9547 images, Atelectasis with 4215 images, Effusion with 3955 images, and Nodule with 2705 images. An overview of the dataset information is provided in Table 5.

## 5. Experimental Results and Data Analysis

The experiments will be divided into multiclass and multi-label types. Some of the terms used will be explained before any further discussion. The attack will be considered a success if “the resulting label is different from the original label”. This applies to both multiclass and multi-label datasets. For the multi-label dataset, if the resulting label contains the original label but with different classes added or deducted, it will still be a success because the resulting label will be “different” from the original one. The “success rate” is used as the index of the attack performance in this study, and the formula is as follows:

(5) $$\;\;\;\;\;\;\;\mathrm{Success}\;\mathrm{rate}=\frac{\mathrm{Number}\;\mathrm{of}\;\mathrm{successful}\;\mathrm{adversarial}\;\mathrm{images}}{\mathrm{Total}\;\mathrm{number}\;\mathrm{of}\;\mathrm{experiments}}\;\;$$

In other words, this is the ratio of the successful experiments compared to the total number of experiments. The number of classes transformed from the original label to another class called the “transform class” will also be counted. Several tables are defined as follows:

- Example of successfully attacked images

Because 100 experiments will be conducted on each class in each dataset, and there will be a large number of successful images if the success rate is high, only one successful image will be presented for each class. The successfully modified image will be shown with a red circle to indicate the modified pixel. There will be two description lines under each image: a bold black line to indicate the original class and a bold red line to show the class that has been transformed. The number in parentheses shows the confidence level of that class. Notably, there will be some confidence levels that indicate “~100%”. This represents a confidence level above 99.94% because the number will be rounded to one decimal point.

- Success rate table

This shows the success rate for each class in the dataset in percentages. The first row is the class name. The second row is the corresponding attack success rate. There will sometimes be an asterisk next to the number in parentheses, indicating that different images were used to construct the attack. Ideally, the algorithm should use 100 different images for the experiment. However, many datasets had too few images to train the model, making the model’s overall accuracy too low to identify some labels or insufficient images were provided to attack that specific class.

- Class transformation table

This is the number of images being transformed for each dataset class. The vertical axis is the original class of the image. The horizontal axis is the resulting class after the transformation. Notably, the axis order is inverted for multi-label datasets, namely the Chest dataset, due to the large number of classes being transformed.

- The ratio of class transformation table

One of the purposes of this study is to explore the resistance of images in each class in the dataset. The class transformation table presented above shows the ratio of class transformation of each class. The ratio can be calculated as follows: Supposing that the original class is denoted as $c_{ori}$ and the adversarial class is denoted as $c_{adv},\;$ there is a total of *K* classes in the dataset, which make $ori,adv\in \left[0,K\right],\;ori,adv,\;K\in N,\;K>1$. The number of transformations from class $c_{ori}$ to $c_{adv}$ is denoted as $n_{ori,adv}$. When ori=adv, the attack is considered to be a failure, so that $n_{ori,adv}=0$. The transformation ratio of the specific adversarial class should be found, which is denoted as $T_{adv}$., which can be calculated as follows:

(6) $$T_{adv}=\frac{\sum_{ori=0}^{K}r_{ori,adv}}{K-F}$$

where $r_{ori,adv}$ is the ratio of transformation from class $c_{ori}$ to $c_{adv}$ and is calculated as follows:

(7) $$r_{ori,adv}=\frac{n_{ori,adv}}{\sum_{adv=0}^{K}n_{ori,adv}}$$

Based on the above formula, $T_{adv}$ is the average effect for each class in $c_{ori}$.

Notably, the formula for $T_{adv}$’s denominator has *F* in it. This denotes the total number of classes that failed the attack. The reason the formula needs to deduct it is that, if one class failed the test, it would not contribute any value on $r_{ori,adv}$ but it would still add 1 to the denominator (consisting of K). Although it will not affect the order of the class transformation ratio, it will lower the total transformation ratio to 100%, which would be incorrect since all transformations should be contained in K classes. Therefore, the effect of the attack ratio can be eliminated using the above formula to obtain a relatively unbiased result of class transformation power.

- Table of class conversion for disease type

This table shows the number and ratio of data converted from “Disease” to “Normal” or “Normal” to “Disease”. The “Class conversion” column indicates whether the type is “Disease to Normal” or “Normal to Disease”. The “Count” column indicates the total images that belong to the conversion type. The “Percentage” column indicates the ratio of the “Count” column.

- Other necessary graphs or tables

Some datasets needed extra figures and tables to further elaborate the results. More will be mentioned and explained as needed.

### 5.1. Multiclass Dataset

(1) Derma

**Figure 2:**
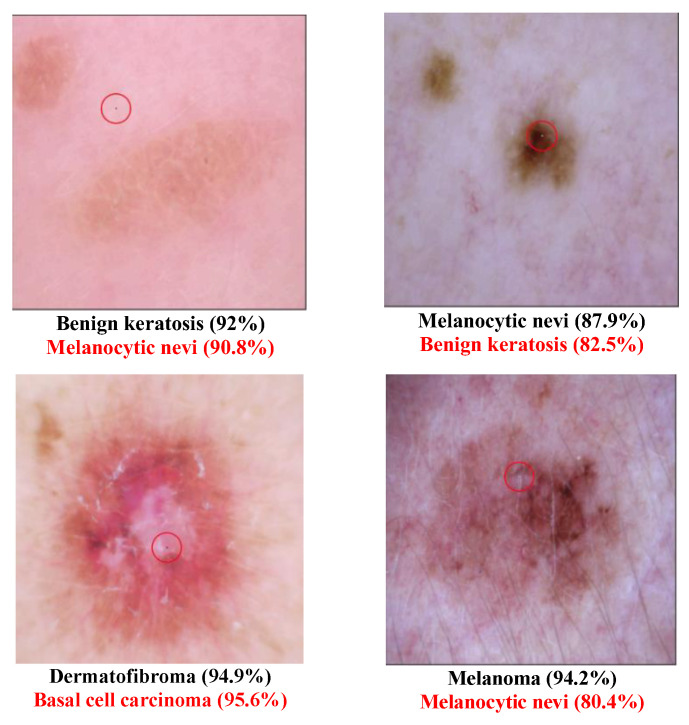
Derma successful attack case examples.

**Figure 3:**
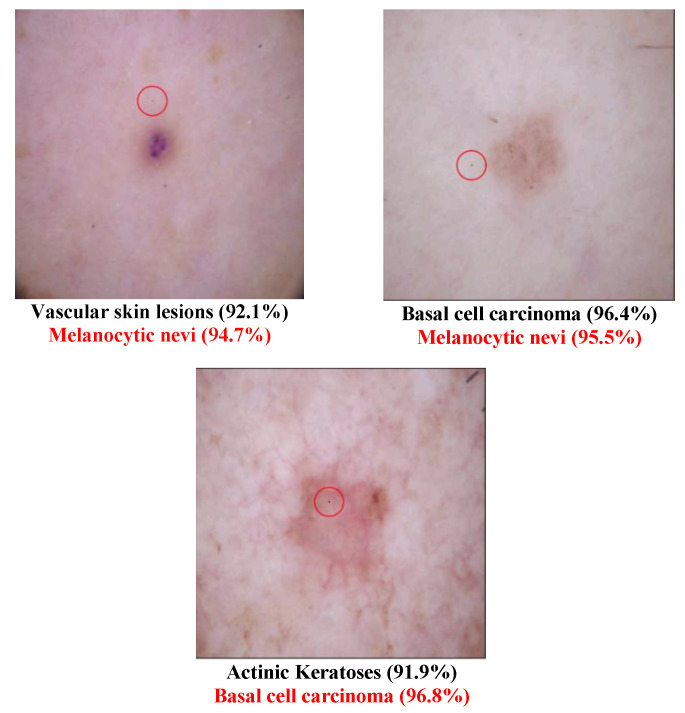
Examples of Successful Derma attacks.

Different Derma class successful attack images are shown in Figure 2 and Figure 3. The class type that has the disease “Melanoma” has been transformed to the normal class “Melanocytic nevi” in Figure 2. This may be because these two types are similar to each other. In Figure 3, several diseased images are categorized into normal images after the one-pixel attack. Table 6 shows that the class Dermatofibroma is more susceptible to attack than other classes. Table 7 lists the class transformation of the Derma database. It is apparent from Table 8 that the top three classes that are more easily transformed are “Melanocytic nevi”, “Basal cell carcinoma”, and “Benign keratosis”. After further analysis of the data, Table 9 shows that it is easier to transform normal images into diseased images than to transform diseased images into normal images.

(2) Pneumonia (Multi-pixel)

The Pneumonia dataset encompasses two different image classes, namely “Normal” and “Pneumonia”. A multi-pixel attack strategy was used on both classes in this research with the objective of transforming the images into their respective opposite categories.

**Figure 4:**
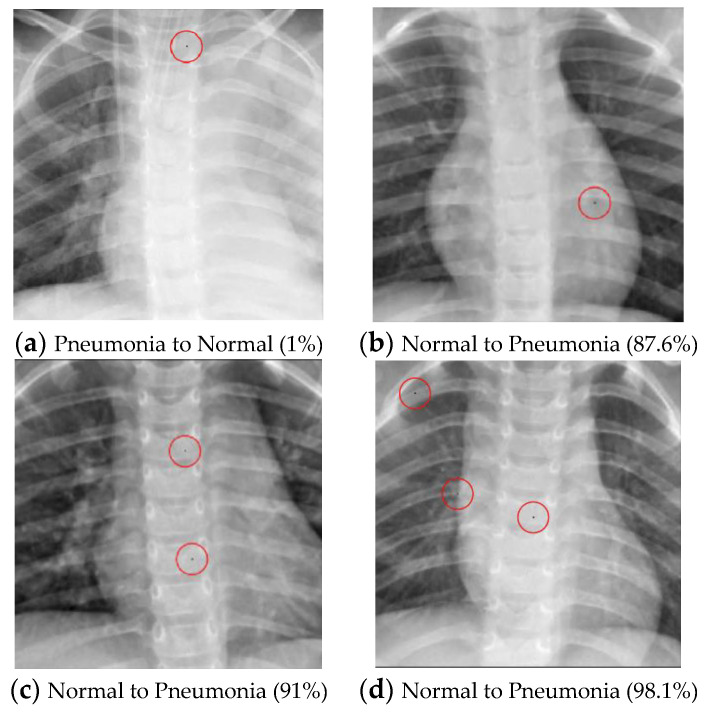
Examples of the attack results on the Pneumonia dataset with confidence level of ResNet50: (**a**) One-pixel attack turning Pneumonia to Normal; (**b**) One-pixel attack turning Normal to Pneumonia; (**c**) Two-pixel attack turning Normal to Pneumonia; (**d**) Three-pixel attack turning Normal to Pneumonia.

As illustrated in Table 10, the success rate of converting normal images into pneumonia images is positively correlated with the number of perturbed pixels. However, the algorithm’s performance in transforming pneumonia images into normal images does not yield successful results, even with an increase in the perturbed pixels. The successful attack images are shown in Figure 4.

(3) OCT (Multi-pixel and target attack)

The OCT dataset comprises four distinctive classes: “Normal”, “Choroidal neovascularization” (CNV), “Diabetic macular edema” (DME), and “Drusen”. Three targeted attacks were conducted, each aiming to transform the images from one of the three disease classes into normal images. The outcomes of these attacks are presented in Table 11. Consistent with the results observed in the pneumonia dataset, the attack that converted normal images into diseased images demonstrated a high success rate by exhibiting a positive correlation with the number of perturbed pixels.

In the targeted attacks, the algorithm struggled to generate successful attacks when attempting to convert CNV and DME images into the normal class using only one or two perturbed pixels. It was not until the number of perturbed pixels was increased to three that the attack began to generate successful transformations.

**Figure 5:**
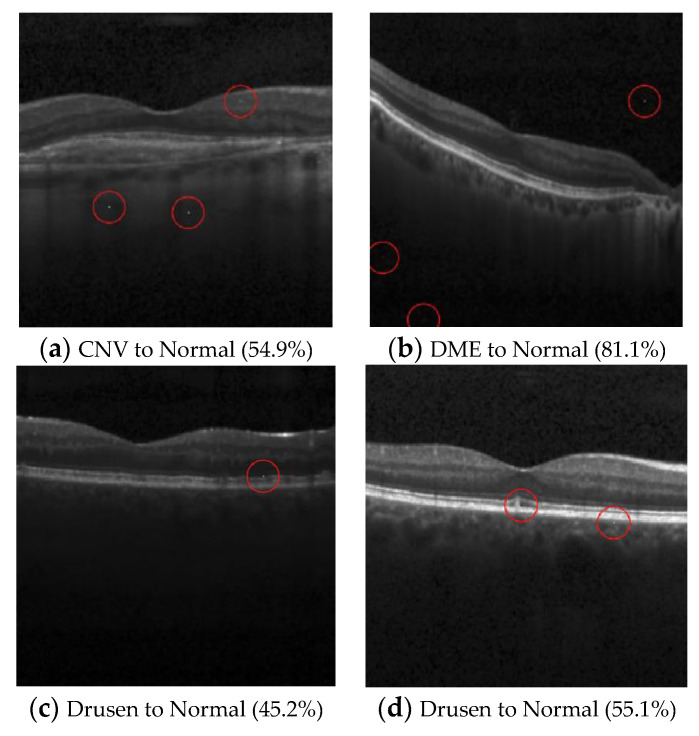
Examples of the attack results on the OCT dataset with confidence level of ResNet50: (**a**) Three-pixel attack turning CNV to Normal; (**b**) Three-pixel attack turning DME to Normal; (**c**) One-pixel attack turning Drusen to Normal; (**d**) Two-pixel attack turning Drusen to Normal.

**Figure 6:**
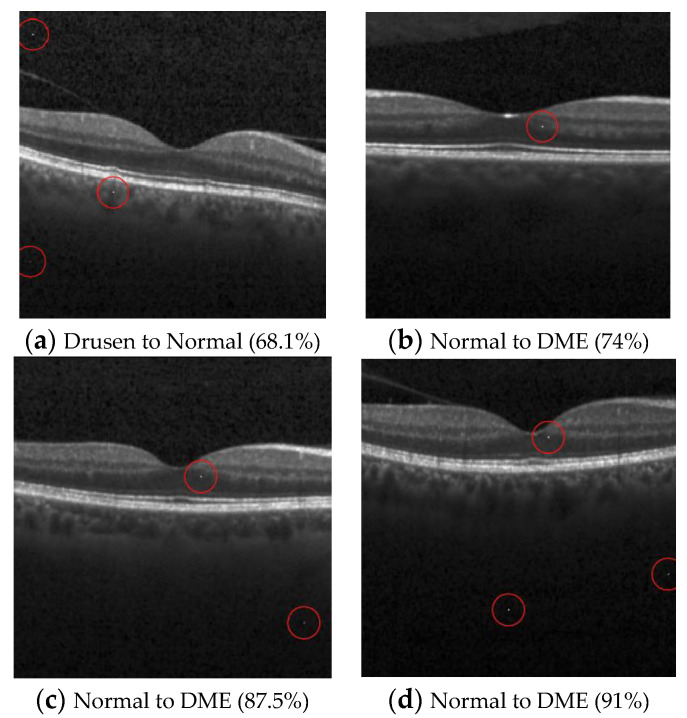
Examples of the attack results on the OCT dataset with confidence level of ResNet50: (**a**) Three-pixel attack turning Drusen to Normal; (**b**) One-pixel untargeted attack on Normal images (c) Two-pixel untargeted attack on Normal images; (**d**) Three-pixel untargeted attack on Normal images.

When converting Drusen images into the normal class, the success rate remained unchanged despite increasing the number of perturbed pixels. This result indicates that the algorithm may find it difficult to adapt to specific classes of images or that the Drusen class exhibits certain characteristics that render the algorithm less effective. The successful attack images are shown in Figure 5 and Figure 6.

(4) COVID-19 (Multi-pixel and target attack)

The average confidence level was used as an additional evaluation metric, alongside the success rate, for the multi-pixel attack in order to better understand the effect of the variation in the number of perturbed pixels on the confidence of the classification.

(8) $$AvgCL=\frac{\sum_{i=1}^{N_{suc}}CL_{i}}{N_{suc}}$$

$N_{suc}\;$ is the number of adversarial samples, and the victim model is classified into the target class. As shown in the above equation, *AvgCL* was the average confidence level of the target class in the adversarial images.

- Diseased images to Normal images

**Figure 7:**
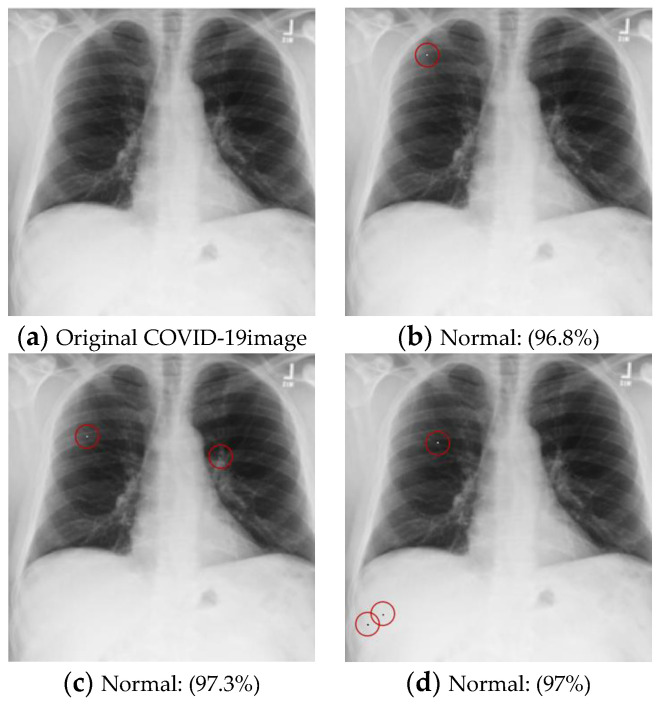
Examples of the attack results on the COVID-19 dataset with confidence level of ResNet50: (**a**) The original COVID-19 image; (**b**) One-pixel attack; (**c**) Two-pixel attack; (**d**) Three-pixel attack.

This dataset contained two diseases: “Pneumonia” and “Covid”. The goal was to perturb a pixel in the original disease image and turn it into a normal image. Since the attacked image had to be classified as “Normal”, this was a targeted attack. The test dataset belonging to “Pneumonia” and “Covid” was attacked, and the results were evaluated based on the Success Rate and Average Confidence Level. A further experiment was conducted on two- and three-pixel attacks to understand the influence of the number of perturbed pixels on the performance of the victim model, and the results are shown in Table 12. The examples of attack images are shown in Figure 7.

Logically, with the increase in perturbed pixels, the value of *AvgCL* should also increase because it changes more parts of the image, making the attack easier.

However, the *AvgCL* was found to have dropped significantly in the three-pixel attack on the “Covid” class. It is believed that this may have been caused by the newly generated three-pixel attack images, which are more difficult to attack so the confidence levels in the target class were also lower. This may have affected the overall *AvgCL*.

- Normal images to Diseased images

An experiment was also conducted using non-target attacks that turn normal images into diseased images to compare it with target attacks that turn diseased images into normal ones. The results can be seen in Table 12. Obviously, it was found to be easier to turn normal images into diseased images.

The class distribution was further examined after the attack in Table 13, and the results show that the proportion of two different disease classes generated is similar, but the *AvgCL* of “Covid” is always higher. This may imply that the victim model tends to “believe” that an image belongs to “Covid”. The result may explain why it is harder to attack “Covid” images.

- Other models

All the above experiments were conducted using ResNet50 [15]. To understand how different models may perform under adversarial attacks, the attack method was also tested using DenseNet121 [20], and the results are shown in Table 14. The success rate was found to have declined slightly in both the “Covid” and “Normal” classes, meaning that the model is more robust under pixel-level attacks. However, the success rate of the class “Pneumonia” had increased, although with a lower *AvgCL*. These results indicate that a larger model like DenseNet121 may be even more susceptible than ResNet50 in some circumstances. Also, the results of pixel-level attacks on the COVID-19dataset can be generalized over different CNN networks.

### 5.2. Multi-Label Dataset

**Figure 8:**
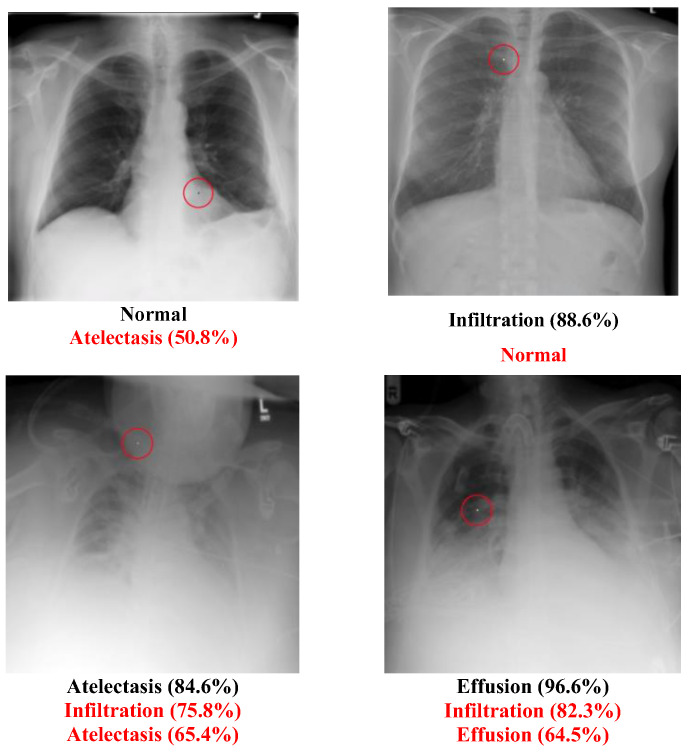
Successful chest attack examples.

**Figure 9:**
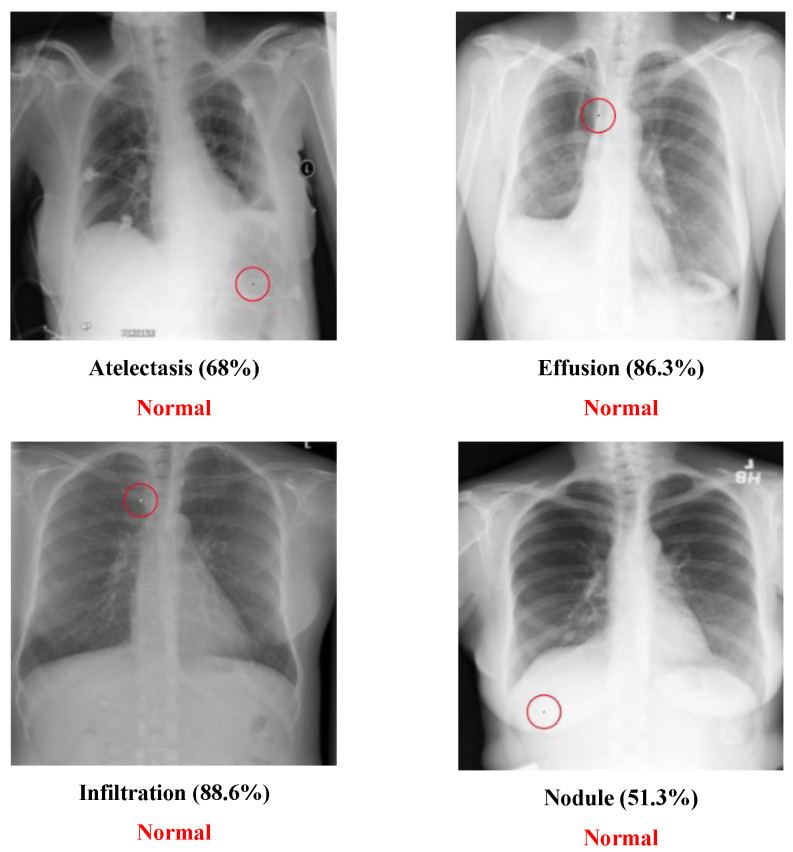
Chest attack cases: Disease to Normal type.

The Chest dataset is a multi-label dataset. Some successful attack examples are shown in Figure 8. The class “Normal” has no confidence level because it means that all labels’ confidence level is below 50%. Figure 9 illustrates some images transformed from a diseased class to a normal class.

**Figure 10:**
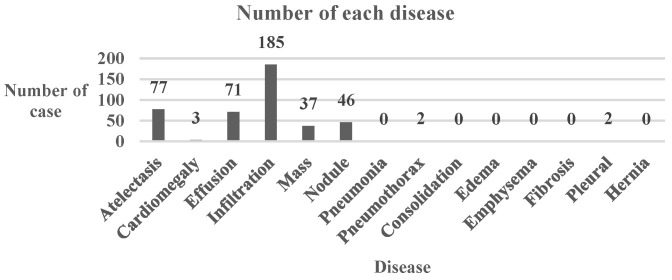
Bar Chart of Number of Each Chest Disease.

It is evident that the “Normal” type had the highest success rate, which is shown in Table 15. The details of the conversion are shown in Table 16. Figure 10 shows the number of diseases in the transformed class of the Chest dataset. The top three were “Infiltration”, “Atelectasis”, and “Effusion”. Table 9 shows that it is easier to transform diseased images into normal ones.

Unlike multiclass datasets, in which the confidence level of the original class needs to be reduced, and the confidence level of other classes increased simultaneously, the confidence level of the original label of multi-label datasets does not need to be reduced too much. The confidence level of other classes only needs to be increased to generate a different label, but when researching the number of disease tables for these two datasets, the final label with one disease is found to be the most common for both datasets. This means that the algorithm will first try to reduce the original class and simultaneously increase another particular disease.

It can be seen from the table of the number of diseases in the Chest dataset that the second most common disease is no disease, which is the normal patient image. This explains why the algorithm will reduce the original label’s confidence level first before trying to increase the other classes’ confidence levels. If reducing the original label has resulted in a different label, which will always be the “Normal” label, the algorithm will stop because it has already reached its goal. Due to this phenomenon, the Chest dataset “Disease to normal” ratio increased dramatically, causing severe concern about the medical system.

In terms of the number of each disease, the types of diseases in the number of diseases table are much the same as the top five labels used in the experiments, meaning that the final table contained fewer rare diseases. This is likely to be because the model is slightly overfitted, so it will tend to predict the image with these labels.

## 6. Discussion

Appendices, if needed, appear before the acknowledgments.

### 6.1. Why Use the One-Pixel Attack?

The major reason for choosing the one-pixel attack is that it is simple and effective. Unlike many other attack methods, it does not rely on gradient descent. This has two main benefits: (1) The problem that needs to be solved does not need to be differentiated, making this attack method more flexible. (2) It requires less computing power and takes less time and cost to implement. Another reason for choosing this attack method is that this kind of “adversarial” image may not be purposely generated. Many other methods that change a lot of pixels in the image to generate adversarial images need time and knowledge, and it is highly likely that the adversarial images will need to be generated purposely. It is possible for adversarial images to be generated naturally using a one-pixel attack. These images may be generated by malfunctioning medical instruments, humans or computers processing images wrongly, or engineers making careless mistakes. They may not be adversarial images that are sufficiently powerful to fool all models, but the goal of a one-pixel attack is not to generate a powerful adversarial image but just a workable one using a reasonable amount of time and computing power.

### 6.2. Comparison of the Colored and Greyscale Datasets

The colored dataset used in the experiment consisted of three RGB channels, whereas the greyscale dataset only contained one channel. More channels can be seen in colored images than in greyscale images, which is likely to affect the time taken for the experiment and the attack success rate. Table 17 shows that the color channel factor had a greater effect on the attack success rate and DE experiment time.

Potential strategies to counter pixel attacks:

- Adversarial Training: Employing adversarial training techniques can enhance the robustness of models by including adversarial examples in the training process. Training the model on both clean and perturbed images makes it more resilient to pixel attacks;
- Input Preprocessing: Input preprocessing techniques such as denoising, image resizing, or blurring can help to remove or reduce the impact of perturbations in the input images, making the model more resilient to pixel attacks; Model Ensemble: An ensemble of multiple models can improve the classification system’s overall robustness. Aggregating the predictions of multiple models can minimize the impact of individual pixel attacks.

**Figure 11:**
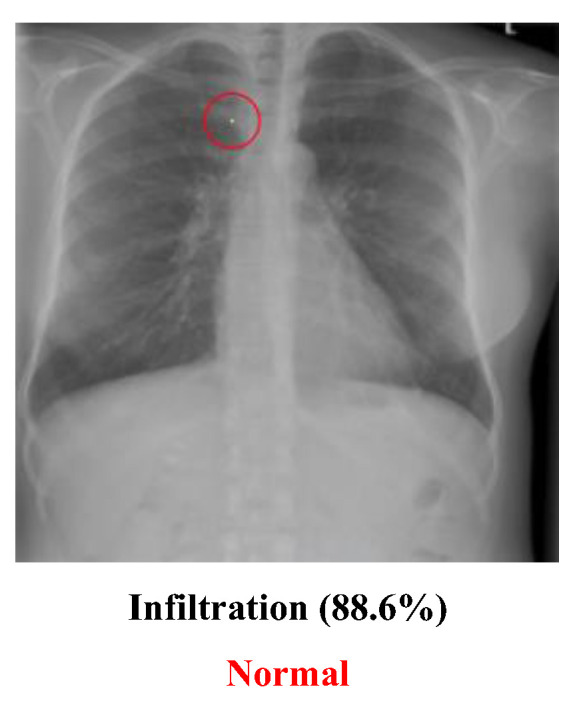
Wrongly labeled image.

Limitations of this study:

Although many experiments were conducted in this study to illustrate that there may be several potential threats when using AI to recognize medical images, it is imperative to understand the severity of the impact of this phenomenon on the medical system. Will successful adversarial images cause the medical system to collapse? A real doctor dedicated to chest diseases was interviewed, and he provided some important advice:

- The medical dataset used for labeling may not be 100% correct. The people who provided the dataset may not have correctly labeled the image, and those who used it may have been unaware of or unable to recognize the mistakes. For example, Figure 11 may not be labeled correctly. Although the image in this dataset is labeled “Infiltration”, which means that the original image had a disease named “Infiltration”, the doctor thought that the image was actually a “Normal” image. He guessed that the lower part of the image might cause the people who labeled the image to think it had been infiltrated, but it may actually be normal muscle cells. The problem of incorrect labeling questions the use of an open-source dataset;

- A one-pixel attack may be capable of generating many adversarial images, but a real doctor will check multiple images with different angles and opacity on one patient. Unless multiple images have been attacked, it may have little impact on the medical system. Also, when doctors make a diagnosis, they do not determine if the patient has a disease by just looking at medical images. They also collect other information, including the patient’s medical records, to assist with their diagnosis. If the doctor thinks the patient may potentially have a disease, but he cannot diagnose it immediately, he may invite the patient to have follow-up treatments. All these measurements in today’s medical system indicate that the treatment may not be disrupted purely by one adversarial image;
- Successful adversarial images were generated in this study with all possible outcomes. These outcomes can be classified by disease type as “Normal to Normal”, “Normal to Disease”, “Disease to Disease”, and “Disease to Normal”. The only type that is of interest to doctors is “Disease to Normal” because “Normal to Normal” will not affect the patient’s life, and “Normal to Disease” and “Disease to Disease” will be more likely to be subject to additional treatment and have a high chance of being re-classified into their correct category. Only “Disease to Normal” may affect the diagnosis.

### 6.3. Future Directions

Although many experiments were conducted in this study using various medical image datasets, many aspects could not be covered due to time and space constraints. The following are some of the aspects that might be considered for further research in the future:

- ResNet50 was used in this study as the basic model being attacked, and some experiments were conducted to illustrate the likely consequences of using different models. However, there are still other neural networks and models that can be used for image identification. For example, capsule-type models [21], Transformers [22], and so on. Another related direction would be to use a real model in the medical domain, such as IBM CODAIT’s MAX breast detector, for comparison;
- Image classification is just one of the tools used in the medical domain. Image segmentation is also a popular type. FGSM was used in a prior study [23] to generate adversarial images for image segmentation tasks on medical images, but a one-pixel attack was not included in the paper. Few researchers have specifically covered the one-pixel attack for medical image segmentation scenarios so far;
- The experiments in this study demonstrated possible weaknesses in several medical image datasets under the one-pixel type of attack. Researchers can use this study to develop an effective way to defend against this attack, specifically for medical image datasets.

## 7. Conclusions

The experiments conducted in this study have shown that nearly all types of medical image datasets, whether greyscale or colored, X-ray, or many other types, can be modified into adversarial images with a one-pixel attack. In addition, multi-pixel attacks were made on the COVID-19 dataset to investigate the impact of the number of perturbed pixels on the success rate and average confidence level. Furthermore, a robust evaluation method was devised for the multi-label Chest dataset to assess the effectiveness of attacks specifically designed for multi-label datasets. The results demonstrated the great risk of using AI or machine learning models to identify medical images and give medical assistance or advice. These experiments showed that adversarial images could result from having no disease to having diseases or vice versa, misjudging the severity of the disease or even classifying the disease type incorrectly. If AI malfunctioned, it would have devastating consequences. Giving the wrong medical advice could delay much-needed treatment, and taking the wrong medicine would cause severe side effects. Medical resources would be wasted, and patients’ lives would be lost in worst-case scenarios. Furthermore, one-pixel attack-like adversarial images could be generated effortlessly, and related knowledge may be assumed mistakenly as a result. In summary, this study provides a major warning of the possible weaknesses of implementing AI in the medical system.

## Figures and Tables

**Table 1:** Overview of the dataset information.

Name	Number of Data	Number of Class	Original Size of Image	Type of Dataset	Color Information	Type of Image
Derma	10,015	7	600 × 450	Multi-class	Color	Dermatoscopic
Pneumonia	5856	2	(384~2916) × (127~2713)	Multi-class	Greyscale	X-ray
OCT	109,309	4	(1, 3) × (384~1536) × (277~512)	Multi-class	Greyscale	Optical Coherence Tomography
COVID-19	30,530	3	1024 × 1024	Multi-class	Greyscale	X-ray
Chest	112,120	14	1024 × 1024	Binary-class Multi-label	Greyscale	X-ray

**Table 2:** Overview of the Derma dataset information.

Class	Count	Percentage	Disease Type	Precision	Recall
Actinic keratoses and intraepithelial carcinoma	327	3.27%	Disease	57%	62%
Basal cell carcinoma	514	5.13%	Disease	65%	74%
Benign keratosis-like lesions	1099	10.97%	Normal	70%	61%
Dermatofibroma	115	1.15%	Normal	67%	35%
Melanoma	1113	11.11%	Disease	68%	56%
Melanocytic nevi	6705	66.95%	Normal	90%	95%
Vascular lesions	142	1.42%	Disease	91%	75%

**Table 3:** Overview of the COVID-19 dataset information.

Class	Count		Percentage		Disease Type	Precision	Recall
	train	test	train	test			
Normal	8085	100	26.83%	25%	Normal	90%	95%
Covid	16,490	200	54.73%	50%	Disease	99%	91%
Pneumonia	5555	100	18.44%	25%	Disease	91%	94%

**Table 4:** Labels of Chest dataset.

Label	Disease	Label	Disease
0	Atelectasis	7	Pneumothorax
1	Cardiomegaly	8	Consolidation
2	Effusion	9	Edema
3	Infiltration	10	Emphysema
4	Mass	11	Fibrosis
5	Nodule	12	Pleural Thickening
6	Pneumonia	13	Hernia

**Table 5:** Overview of the Chest dataset information.

Disease count	Count	Percentage	Disease Type
Normal	11,928	53.17%	Normal
1	6259	27.90%	Disease
2	2914	12.99%	Disease
3	989	4.41%	Disease
4	275	1.23%	Disease
5	54	0.24%	Disease
6	12	0.05%	Disease
7	2	0.01%	Disease

**Table 6:** Derma success rate.

Class	Benign Keratosis	Melanocytic Nevi	Dermatofibroma	Melanoma	Vascular Skin Lesions	Basal Cell Carcinoma	Actinic Keratoses
Success rate	41%	4%	80%	16%	10%	32%	41%

**Table 7:** Derma class transformation.

	Transform	Benign Keratosis	Melanocytic Nevi	Dermatofibroma	Melanoma	Vascular Skin Lesions	Basal Cell Carcinoma	Actinic Keratoses
Original								
Benign Keratosis			18	1	8	0	9	5
Melanocytic Nevi		3		0	0	0	1	0
Dermatofibroma		2	18		0	0	57	3
Melanoma		4	11	0		0	1	0
Vascular Skin Lesions		0	9	0	0		1	0
Basal Cell Carcinoma		7	12	4	3	0		6
Actinic Keratoses		10	0	4	3	0	24	

**Table 8:** Derma ratio of class transformation.

Class	Benign Keratosis	Melanocytic Nevi	Dermatofibroma	Melanoma	Vascular Skin Lesions	Basal Cell Carcinoma	Actinic Keratoses
Transform ratio	21.25%	37.52%	3.53%	5.17%	0.00%	27.57%	4.96%

**Table 9:** One-Pixel Class conversion type.

Dataset	Class Conversion	Count	Percentage
Derma	Disease to Normal	61	42.36%
	Normal to Disease	83	57.64%
Chest	Disease to Normal	156	62.15%
	Normal to Disease	95	37.85%

**Table 10:** Pneumonia: Attack Results.

ResNet50	One-Pixel	Two-Pixel	Three-Pixel
Normal	64%	66%	70%
Pneumonia	0%	0%	0%

**Table 11:** OCT: Attack Results.

ResNet50	One-Pixel	Two-Pixel	Three-Pixel
Normal (untargeted)	62%	66%	69%
CNV to Normal	0%	0%	1%
DME to Normal	0%	0%	1%
DRUSEN to Normal	3%	3%	3%

**Table 12:** COVID-19: Results of RESNET-50.

ResNet50		One-Pixel	Two-Pixel	Three-Pixel
Pneumonia	*SR*	4%	5%	4%
	*AvgCL*	0.836	0.857	0.915
Covid	*SR*	1.5%	2%	2.5%
	*AvgCL*	0.718	0.720	0.653
Normal to Disease	*SR*	21%	23%	22%
	*AvgCL*	0.835	0.830	0.826

**Table 13:** COVID-19: Normal Images to Diseased Images.

ResNet50	One-Pixel		Two-Pixel		Three-Pixel	
	Covid	Pneumonia	Covid	Pneumonia	Covid	Pneumonia
Proportion	57%	43%	48%	52%	45%	55%
AvgCL	0.878	0.778	0.887	0.778	0.862	0.796

**Table 14:** COVID-19: Results of DenseNet121.

DenseNet121		One-Pixel	Two-Pixel	Three-Pixel
Pneumonia	*SR*	4%	6%	7%
	*AvgCL*	0.578	0.616	0.630
Covid	*SR*	1%	1.5%	2%
	*AvgCL*	0.711	0.671	0.603
Normal to Disease	*SR*	18%	17%	16%
	*AvgCL*	0.717	0.705	0.738

**Table 15:** Chest success rate.

Class	Normal	Infiltration	Atelectasis	Effusion	Nodule
Success rate	95%	85%	94%	87%	91%

**Table 16:** Chest class transformation.

	Original	Normal	Infiltration	Atelectasis	Effusion	Nodule
Transform						
Atelectasis		13	3	N/A	3	5
Atelectasis, Effusion		0	1	7	15	0
Atelectasis, Effusion, Infiltration		0	0	1	0	0
Atelectasis, Infiltration		2	9	15	2	0
Atelectasis, Nodule		0	0	0	0	1
Cardiomegaly		1	1	1	0	0
Effusion		8	1	6	N/A	4
Effusion, Infiltration		1	4	0	19	0
Effusion, Mass		0	0	0	1	1
Effusion, Mass, Pleural		0	0	0	2	0
Infiltration		51	N/A	17	13	17
Infiltration, Edema		0	6	0	0	0
Infiltration, Nodule		2	5	0	0	21
Mass		13	1	2	2	0
Mass, Nodule		1	0	0	0	14
Nodule		2	0	0	0	N/A
Normal		N/A	54	44	30	28
Pneumothorax		1	0	1	0	0

**Table 17:** Color channel analysis.

Color Channel	Average Attack Success Rate	Average DE Time
Grayscale	19%	2 min 49 sec
Colored	25%	3 min 07 sec

## Data Availability

All data are publicly available.
